# Polyurethane Foam as a Novel Material for Ochratoxin A Removal in Tea and Herbal Infusions—A Quantitative Approach

**DOI:** 10.3390/foods12091828

**Published:** 2023-04-28

**Authors:** María del Valle Ponce, Mariel Cina, Carlos López, Soledad Cerutti

**Affiliations:** 1 Instituto de Química de San Luis (INQUISAL-CONICET-UNSL), Laboratorio de Espectrometría de Masas, Facultad de Química Bioquímica y Farmacia, Universidad Nacional de San Luis, Bloque III, Ejército de los Andes 950, San Luis 5700, Argentina; 2 Consejo Nacional de Investigaciones Científicas y Técnicas (CONICET), Av. Rivadavia 1917, Buenos Aires 1033, Argentina; 3 Instituto de Investigaciones en Tecnología Química (INTEQUI-CONICET-UNSL), Facultad de Química Bioquímica y Farmacia, Universidad Nacional de San Luis, Almirante Brown 1455, San Luis 5700, Argentina

**Keywords:** Ochratoxin A, polyurethane foam, infusions, UHPLC-MS/MS

## Abstract

A novel solid-phase extraction methodology followed by UHPLC-MS/MS has been developed for Ochratoxin A (OTA) analysis in herbal infusions. For this purpose, a commercial polyurethane foam (PUF) was used as sorbent, and the experimental conditions were fully optimized. The strategy was satisfactory for reducing the matrix effect and allowed for OTA quantification in black tea and herbal infusions, with suitable recoveries and quantitation limits in agreement with those required by the maximum levels allowed by current regulations. The achieved results demonstrated the unprecedented use of polyurethane foam as an effective alternative for OTA retention and quantification in herbal infusions with the advantages of simple preparation, time saving, sustainability, and low cost for routine analysis.

## 1. Introduction

Tea is one of the most widely consumed beverages after water, with worldwide production and consumption of approximately three billion kilograms per year [1,2]. The FAO’s reports (Food and Agriculture Organization of the United Nations) stated a growth over the last decade of 2.5% of tea ingestion per capita accompanied by strong market expansions in the producing countries [3]. Two main categories of tea can be defined: tea and herbal tea. The term “tea” refers to the beverage obtained from hot water and the *Camellia sinensis* plant. Depending on the process on the plant, there are several types of teas, including white, green, oolong, black, and pu-erh teas [4]. On the other hand, “herbal tea” is a beverage made with hot water and fruits, flowers, leaves, stems, and roots of a variety of plants that do not contain *C. sinensis*. Some of the most popular herbal teas (also known as herbal tisanes or herbal infusion) are ginger tea, peppermint tea, and chamomile tea. In addition, within this category of herbal infusion is Mate tea, a variation of the traditional Mate (*Ilex paraguariensis)*, which is very popular in South America [5,6].

Despite the value of tea drinking for human health (as a main source of nutrients and also due to its antioxidant [7], cardioprotective [8], anti-inflammatory [9], anti-obesity [10], and anti-cancer properties [11]), a cup of tea may contain contaminants such as pesticides, toxic heavy metals, environmental pollutants, microorganisms, mycotoxins, among others that can threaten consumers’ health seriously [12].

Mycotoxin is a term that includes a wide variety of secondary metabolites produced by molds. While more than 400 mycotoxins have been identified, the main toxins affecting agriculture, economics, and public health aspects are aflatoxins, ochratoxins, fumonisins, trichothecenes, and zearalenone [13].

Ochratoxin A (OTA) is one of the most harmful mycotoxins produced by some species of *Penicillium* sp. and *Aspergillus* sp. fungi [13]. Many toxic effects of OTA have been reported as a result of several studies carried out on its carcinogenic properties. The International Agency for Research on Cancer classified OTA as a potential human carcinogen (category 2B) [14]. This toxin is a contaminant in a wide number of foodstuffs as cereals, pulses, pumpkin seeds, cocoa products, wine, beer, coffee, and tea [13], and has been monitored and regulated in the last two decades. Recently, the Commission Regulation (EU) [15] has established the maximum contents of OTA in dried herbs, with a maximum level allowed of 10 ng mL^−1^. The popularity of these beverages increases the risk of dangerous chronic low-dose exposure to OTA. Consequently, the development of analytical methods that allow for the reliable determination of this toxin in beverages at levels according to the current regulations represents a growing field of study [16].

Several analytical methods have been developed for the analysis of OTA in food and beverages. The techniques most used are generally based on enzyme-linked immunosorbent assay (ELISA) or chromatographic techniques, such as liquid chromatography (LC), thin-layer chromatography (TLC), and gas chromatography (GC) [13,17]. Moreover, besides the natural fluorescence of OTA, the advances in analytical instruments have become high-performance liquid chromatography coupled with mass spectrometry (HPLC-MS) as the gold standard method for mycotoxins detection [18].

During the analysis of food and beverage samples, the preparation step is a critical stage when the elimination or minimization of interference, the separation, and enhancement of analytes, or the accomplishment of compatibility with the determination technique are required [19]. Classical extraction techniques include solid-phase extraction (SPE) and liquid–liquid extraction (LLE) approaches. Nevertheless, these conventional methodologies involve many steps, time consumption, and the use of a high quantity of toxic solvents [20]. The new trends in Green Analytical Chemistry (GAC) demand more environmentally friendly protocols [21], and consequently, novel sample preparation techniques have been developed. The solid-phase microextraction (µSPE) [22], dispersive solid-phase extraction (d-SPE) [23], and dispersive liquid–liquid microextraction (d-µLLE) [24] represent some examples of this progression to greener methodologies. In this sense, the evaluation of several materials such as MIP (molecularly imprinted polymers), MOF (metal-organic frameworks), and COF (covalent organic frameworks) have been reported to improve methodological performance, while the environmental impact is reduced [20]. Although these techniques are demonstrated to be suitable for the simultaneous determination of herbal infusions constituents, the previously mentioned weaknesses make these strategies unsuitable for rapid and routine evaluation of these drinking beverages’ quality [25].

Polyurethane foam (PUF) represents an excellent solid sorbent material for solid-phase extraction [26]. The chemical structure of PUF contains a high concentration of polar and nonpolar sections, which allows them to retain varied analytes. Since 1970, when the first report on the use of this plastic material for sorption experiments was published [27], it has been used for the separation and recovery of several trace compounds from aqueous solutions [28,29,30,31]. The increasing attention on the use of PUF’s for removal purposes is due to their unique properties, such as high surface area, open cellular structure, chemical resistance, safety, reusability, and low-cost manufacturing.

According to the above-mentioned, this work aimed to develop a new, rapid, low-cost, and eco-friendly PUF-based extraction methodology to remove OTA from commercial tea and herbal infusions followed by chromatographic and spectrometric analysis. In addition, the greenness assessment of the optimized procedure was carried out through several metrics, obtaining satisfactory results. The optimized overall methodology allowed us to obtain analytical capabilities compatible with the current regulatory demands.

## 2. Materials and Methods

### 2.1. Chemicals and Reagents

Water Optima^TM^ (H_2_O), methanol (MeOH), and acetonitrile (ACN) were LC/MS grade from Fisher Scientific (Fair Lawn, NJ, USA). The analytical standard for OTA (*Petromyces albertensis* with a purity higher than 98.0%) was obtained from Fluka (Steinheim, Germany). Standard stock solutions were prepared in MeOH and stored at −20 °C. Ultrapure water was obtained with a Milli-Q system (Millipore Corporation, RF Barnstead, IA, USA). Formic acid (FA) and acetic acid (AA) were purchased from Fisher Scientific (Fair Lawn, NJ, USA), while ammonium formate and ammonium bicarbonate were from Fluka (Steinheim, Germany).

### 2.2. UHPLC-MS/MS Conditions

As in previously developed works by our group, the equipment consisted of an ultra-high performance liquid chromatograph (Waters, Milford, CT, USA) associated with a triple quadrupole mass spectrometer (Quattro Premier ™ XE Micromass MS Technologies), configured with electrospray ionization source (ESI (+), Z-Spray ™, Waters, Milford, CT, USA). Full scan and fragment ion experiments were performed using the following parameters: ion spray voltage of 3.5 kV, extractor voltage of 1.0 kV, desolvation gas (ultrapure N_2_) at 800 L h^−1^, collision gas (Ar) at 0.18 mL min^−1^, nebulizer, source temperature of 150 °C, and desolvation temperature of 350 °C. Multiple reaction monitoring (MRM) mode was selected. The quantification transition, 404.1→239.2, was produced at a collision energy of 25 eV, and the confirmation fragments, 404.1→341.1 and 404.1→358.2, at collision energies of 25 and 20 eV, respectively. The chromatographic resolution was performed using a reverse-phase C18 column (50 mm × 2.1 ID × 1.7 μm, Waters, Milford, USA), conditioned to a temperature of 35 °C. The elution was conducted in the isocratic mode using 0.1% (*v*/*v*) formic acid in water (A) and 0.1 % (*v*/*v*) formic acid in acetonitrile (B). The solvents were programmed as follows: 10% A and 90% B, pumped at a 0.25 mL min^−1^ flow rate. The sample injection volume was 10 μL. Under these conditions, the retention time for OTA was 0.7 min. Data acquisition was conducted using the MassLynx Mass Spectrometry software (Waters, Milford, CT, USA).

### 2.3. Preparation and Characterization of Sorbent

Flexible polyurethane foam (PUF) commercially available by Make Argentina was chopped into minor pieces. Then, the solid material was washed with nitric acid (30% *v*/*v*), followed by methanol, and finally, several times with ultrapure water until neutral pH was obtained. Then, the PUF was dried on a stove at 80 °C for 1 h. A scanning electron microscope (SEM), LEO 1450VP EDAX microscope, was used to analyze the surface morphology of PUF, from which a highly porous structure with a minimal impediment to solvent flow and a high surface area for the retention of compounds of interest was observed.

### 2.4. Extraction and Clean-Up Procedure

Experiments were carried out in batch systems, and the clean-up procedure involved two stages. The first step involved the extraction of OTA from the aqueous solution. For this purpose, a 3 mL analyte’s spiked sample aliquot, at several concentration levels (0, 5, 10, 15, 25, and 50 ng mL^−1^), previously acidified with 25 mM of acetic acid, was added into a Falcon tube containing 50 mg of PUF. The extraction process was assisted by orbital shaking for 10 min at 25 °C. When the contact time ended, the supernatant was discarded. Afterward, the second part of the process comprised the recovery of OTA by using a suitable elution solvent. In this case, the solvent consisted of a mixture of methanol and water, with a mixing ratio of 80:20 (*v*/*v*) and 0.75 % (*v*/*v*) of formic acid. Thus, 3 mL of this elution solvent was added into the falcon tube and shaken at 25 °C for 10 min. Finally, the supernatant was collected and analyzed by UHPLC-MS/MS. Concerning improving the clean-up procedure performance, a two-level three-factors full factorial design consisting of 11 experiments was performed. The studied variables were the mass of PUF (10 to 50 mg), shaking time (10 to 30 min), and sample volume (3 to 5 mL). Analysis of variance (ANOVA) and *p*-value (probability test) were used to evaluate the statistical significance of the effects. The data analysis was performed using the software Minitab Statistic Software 15.1.20.0 (Minitab Inc., State College, Pennsylvania).

### 2.5. Sample Preparation

Commercial samples of tea and herb infusions (black tea, green tea, chamomile tea, and mate tea) were purchased in the local supermarkets (San Luis, Argentina).

For each infusion, 200 mL of ultrapure water was heated to 80 °C, and a bag (1 g) of the dried herb was placed into the glass beaker and left to infuse for 5 min with occasional stirring, according to a procedure previously reported [32].

### 2.6. Matrix Effect and Recovery Essays

Suppression matrix effects (MEs) were calculated with the slope ratio approach proposed by Romero-González et al. and Sulyok et al. [33,34], preparing spiked samples at different calibration levels (0, 5, 10, 15, 25, and 50 ng mL^−1^) in triplicates. According to Equation (1), the overall ME (%) was obtained from the slopes ratios of calibration curves in liquid standard (*a*) and in matrix-matched standard (*b)*.

(1) $$ME\left(\%\right)=\left(1-\frac{b}{a}\right)*100$$

Recovery experiments were performed by both external matrix-assisted calibration and spiked addition. Six-point calibration curves were prepared (0, 5, 10, 15, 25, and 50 ng mL^−1^) in triplicates using blank samples according to the above-described procedure. The amount of OTA recovered was calculated by comparing the determined concentrations of the analyte (*C*) with the theoretical values (*C*_0_), according to Equation (2).

(2) $$RE\left(\%\right)=\frac{C}{C_{0}}*100$$

## 3. Results and Discussion

### 3.1. Optimization of Sample Treatment Conditions

The experimental variables related to the clean-up procedure were optimized for both steps: the retention of OTA by PUF from the aqueous solution and the elution of this mycotoxin using a compatible UHPLC-MS/MS solvent. These experiments were carried out using OTA-spiked solutions at different concentrations under several conditions of pH (from 3 to 9), the mass of PUF (10 to 50 mg), shaking time (10 to 30 min), sample volume (3 to 5 mL), and type of eluent (water, methanol, acetonitrile, ethyl acetate (EtAc), dichloromethane (DCM), and their mixtures.

#### 3.1.1. Optimization of the Extraction Procedure

**Figure 1:**
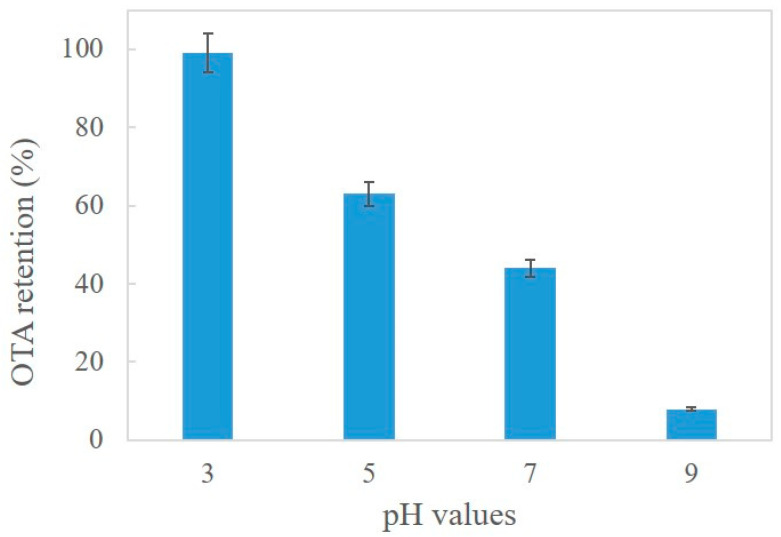
Influence of the pH solution on the PUF absorption capacity of OTA.

OTA (C_20_H_18_ClNO_6_; molecular weight 403.8 g moL^−1^) is an ionizable analyte with two pKa values due to the presence of a carboxyl group and a phenolic hydroxyl group. For this reason, OTA can be found in protonated, nonionic, monoanionic (OTA^−^), and dianionic (OTA^2−^) forms depending on the media conditions. Consequently, the pH value can be of fundamental importance in the separation/extraction process [35]. Thus, the pH influence, from 2 to 9 values, on OTA uptake by PUF, after a 30 min shaking time, was studied. Different buffer solutions (5 mL) were used: acetic acid, ammonium formate, and ammonium bicarbonate. The results in Figure 1 showed a retention efficiency improvement at lower pH values. Despite OTA being partially anionic at lower pH values, the resulting retention in acidic media is probably associated with solvent extraction and/or weak base anion exchange mechanisms. The reported value of the point of zero charges (PZC = 7.6) of PUF indicates that at pH under the PZC, the surface of PUF is positively charged due to the protonation of chelating sites, resulting in enhanced retention of the analyte, as mentioned by other authors [29]. Therefore, the subsequent experiments were performed at pH values of 3–3.5 by using acetic acid.

Other relevant variables, such as sample volume, sorbent mass, and contact time, were optimized with a full factorial design with three factors in two levels (2^3^), resulting in a total of eight runs and three replicates of the central point. The three factors were studied at minimum, central, and maximum points, as shown in Table 1, and the response was OTA retention (%), which was obtained from the chromatographic peak areas. The analysis of the results was performed using Minitab Statistic Software (State College, Pennsylvania).

**Figure 2:**
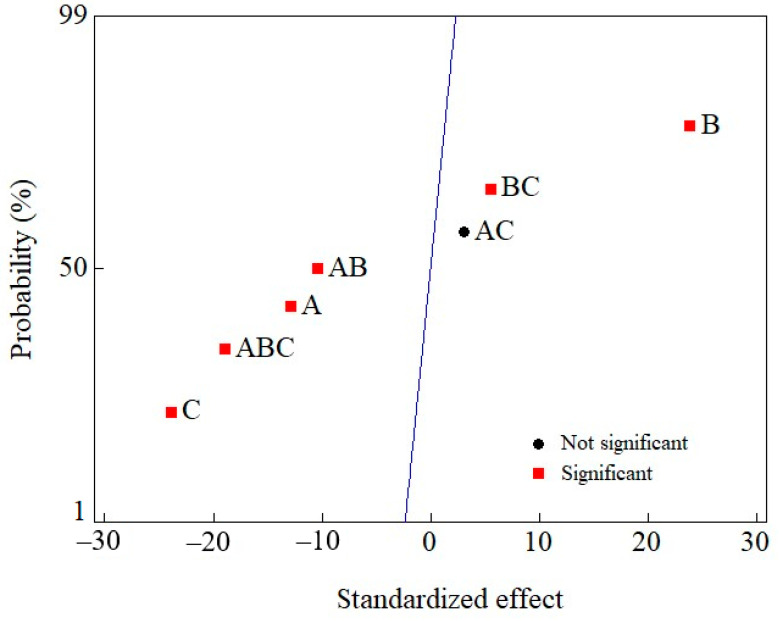
Normal plot of standardized effects (α = 0,05) for sample volume (A), sorbent mass (B), and C (contact time). AB, AC, ABC, and BC refer to the variables’ interactions.

After application, the evaluated variables demonstrated to be statistically significant at the 95% confidence level (Figure 2). Furthermore, sample volume and contact time showed negative standardized effects, indicating that lower values of these factors yielded a higher OTA retention, while the sorbent mass presented the opposite effect, meaning that at a higher sorbent mass, the analyte’s retention efficiency was improved.

Consequently, after further statistical evaluation, a sample volume of 3 mL, a PUF mass of 50 mg, and a contact time of 10 min were chosen as optimal conditions for the first stage of the retention/clean-up procedure. As stated previously, the solution pH was kept constant at the value 3–3.5 by using acetic acid.

#### 3.1.2. Optimization of OTA Elution

**Figure 3:**
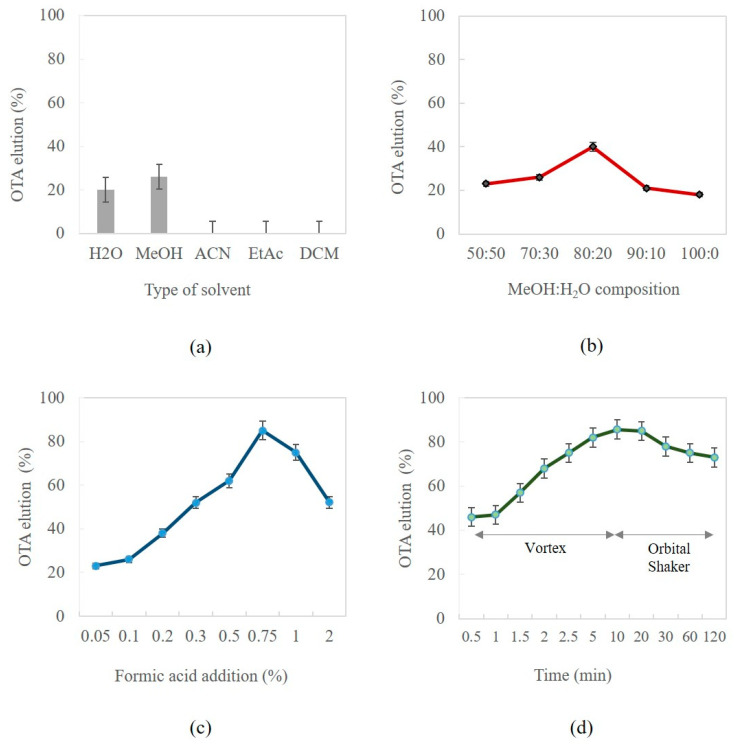
Assessment of elution conditions: (**a**) type of solvent; (**b**) mixture composition (methanol: water); (**c**) formic acid addition; (**d**) time and type of agitation.

The first variable optimized for the elution step was the type of solvent. Therefore, several assays were carried out using water, methanol, acetonitrile, ethyl acetate (EtAc), and dichloromethane (DCM). The results obtained with these solvents and their mixtures showed that methanol and water were the only solvents in which OTA was quantitatively eluted (Figure 3a). Moreover, it was observed that the mixture of them was more efficient than the use of pure solvents. Subsequently, the composition of the mixture MeOH/H_2_O (*v*/*v*) and the effect of its acidification were evaluated. According to what is shown in Figure 3b,c, the composition 80:20 (*v*/*v*) of MeOH/H_2_O, with 0.75% (*v*/*v*) of formic acid, helped to reach the highest OTA elution percentage, between 80% and 90%. Finally, under these conditions, the time and type of agitation assistance were evaluated considering shorter times with stronger agitation (Vortex) and higher times with the same orbital shaker used before. As can be seen in Figure 3d, the elution of OTA was achieved in a few seconds using vortex systems, while with orbital shaking, the best elution condition was obtained at 10 min. However, considering that the orbital shaking allowed for the processing of many samples simultaneously with a suitable consumption of time, it was preferred over vortex stirring. As a result, these conditions were selected to complete the approach.

### 3.2. Matrix Effect and Recovery

**Figure 4:**
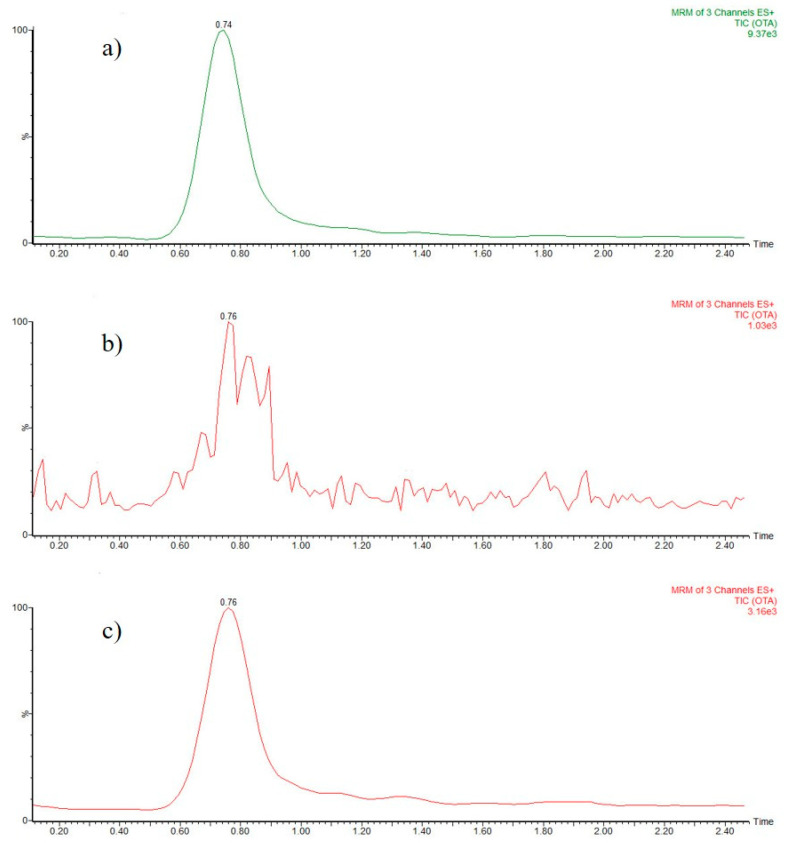
Chromatograms for (**a**) OTA standard (10 ng mL^−1^) in pure elution solvent (80:20 (*v*/*v*) of MeOH/H_2_O with 0.75% (*v*/*v*) of formic acid; (**b**) spiked sample (black tea) of OTA (10 ng mL^−1^) without applying the clean-up step; (**c**) spiked sample (black tea) of OTA (10 ng mL^−1^) after the application of the proposed methodology.

Matrix effects (MEs) caused by the presence of complex sample compounds can enhance or suppress responses from the desired analytes during the ionization process in routine UHPLC-MS/MS analyses, which leads to inaccurate results [36]. After evaluation, the ME in the OTA signal for black tea samples is shown in Figure 4. Thus, a narrow chromatographic peak in the pure solvent elution can be observed (Figure 4a), while in tea samples, without PUF treatment, that peak was not visible or suppressed, due to the presence of other components of the matrix. This situation affected the quantification limits for OTA, and under these conditions, it would be impossible to determine the mycotoxin at or below the maximum allowed levels in dried herbs (10 ng mL^−1^). The use of a simple and fast clean-up methodology, based on a PUF sorbent, significantly reduced the matrix effect and made possible the quantification of OTA at trace levels, as can be seen in Figure 4c.

The matrix effects of the different sample types (black tea, green tea, chamomile tea, and mate tea) were calculated according to the equation given in Section 2.6. The ME (%) values were expressed indicating the observed effect, which in this work was signal suppression. The obtained results were classified according to (i) no matrix effect for values considering <20%, (ii) a medium effect with values 20% < MEs < 50%, and (iii) a strong effect for values higher than 50%. As shown in Table 2, the use of the proposed clean-up methodology allowed for a significant decrease in the matrix effect to medium or low levels in all infusions studied. This information was considered for further quantification purposes.

The extraction recoveries (RE, %) of OTA in herb infusion were measured in samples spiked at different OTA concentrations (from levels closer to the LOD up to 100 ng mL^−1^), particularly considering the level of 10 ng mL^−1^ of OTA, which corresponds to the maximum allowed limit by the European Commission Regulation No. 1370/2022 for dried herbs. The results obtained (Table 2) demonstrated almost 90% of recovery for black, green, and chamomile tea, and nearly 80% for the mate tea infusion, all of them with suitable relative standard deviation (RSDs). Furthermore, the obtained figures of merit showed proper linearity (R^2^ 0.997), linear range, and limits of detection and quantification following those required by the regulation.

### 3.3. OTA Determination in Real Tea Samples

The applicability of the PUF-based method to determine OTA was demonstrated through the analysis of tea infusions made from different tea varieties and prepared in triplicate (n = 3). From the obtained results, it is possible to conclude that the concentration of OTA was below the limit of quantification of the proposed methodology and, therefore, below the regulated limits. Fluorescence detection was also performed on the samples, and no statistical differences were obtained from the results described.

### 3.4. Assessment of Green Sample Preparation

The negative consequences of chemical activities in the environment and the growing awareness for reducing their ecological effects are the main reasons for the research and development of greener analytical methods for sample preparation [37]. From this perspective, following the principles of green chemistry (GC) and more specifically the 12 principles of green analytical chemistry (GAC), the analytical methodologies have gradually improved to greener practices. In recent years, the attention to the sample preparation step has become more significant, and as a result, the principles of GAC have been revised. A new concept of greenness assessment related directly to sample preparation emerged with the 10 principles of green sample preparation (GSP) as a new empowerment tool for the practice of greener protocols [38].

For analytical procedures, the sample treatment is a critical step when the elimination or minimization of interference and/or the separation and enhancement of target analytes are required to achieve the quantification and detection limits in the analysis of trace compounds. In addition, the use of sensitive equipment usually requires the sample preparation stage to ensure agreement with the determination technique [39]. Nevertheless, despite the importance of this stage in the analytical process, sample preparation has been recognized as one of the most critical steps from the GAC perspective. The first principle of green analytical chemistry recommends the elimination of the sample preparation and the use of a direct analytical technique, but this option is rather limited, and a preparation approach is commonly needed [38].

With the purpose of evaluating the greenness of analytical procedures, several tools have been reported. The most usual strategies used for this aim include several metrics, such as National Environmental Methods Index (NEMI) [40], Analytical Eco-Scale (AES) [41], Green Certificate [42], Green Analytical Procedure Index (GAPI) [43], RGB additive color model (RGB) [44], Analytical GREEnness (AGREE) [45], Complementary Green Analytical Procedure Index (ComplexGAPI) [46], and later introduced, Analytical Greenness Metric for Sample Preparation (AGREEprep) [39]. Most of them are based on the 12 principles of GAC, except the for AGREEprep metric, which is based on the 10 principles of GSP. Additionally, some of these metrics possess free software to facilitate their application.

In this work, the Green Certificate, AGREE, and AGREEprep were applied to assess the proposed PUF-based sample preparation methodology.

In this sense, the Green Certificate is a modified analytical eco-scale for the quantification of the greenness of a process. Some variables such as reagent toxicity, energy consumption, and waste generated in the analysis are considered. From the assessment of these aspects, the penalty points are obtained (in a similar way to the eco-scale) and subtracted to an ideal score of 100 [47]. In addition to the number, a color code linked with a letter from A to G is used. The A classification is obtained with less than 10 penalty points (the greenest one), the B class method discounts between 11 and 20 penalty points, the C classification, from 21 to 30 penalty points, the D class between 31 and 45 points, the E class sums between 46 and 60 penalty points, the F class between 61 and 80, and finally, the G class considers more than 81 penalty points, being the least green method.

**Figure 5:**
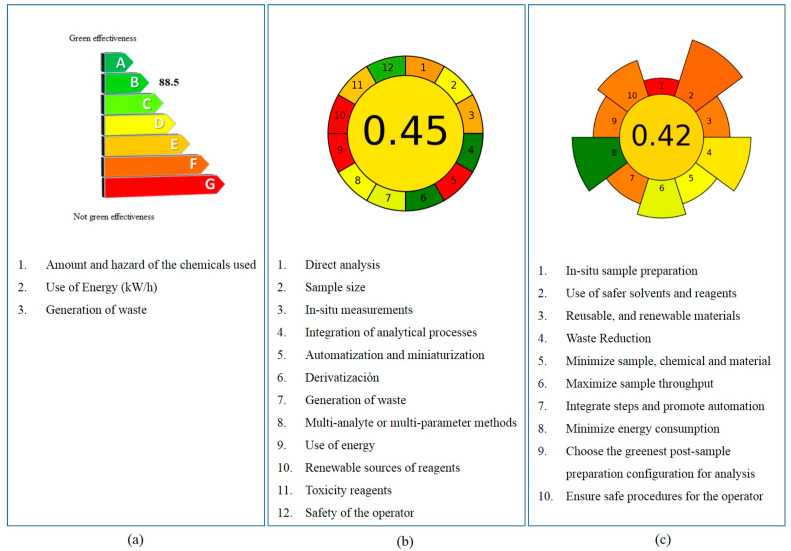
Criteria and results of greenness evaluation of the proposed sample preparation method using different approaches: (**a**) Green certificate, (**b**) AGREE, and (**c**) AGREEprep metrics.

As can be seen in Figure 5a, the proposed sample preparation was classified as B class, with a score of 88.5 points. Most of the penalty points subtracted were associated with the hazard of solvents and the quantity of generated waste. However, to overcome the fact that this result does not provide further information about the nature of threats, other metrics were applied.

The evaluation of analytical procedures using the Analytical GREEnness calculator (AGREE) is based on the application of the 12 principles of green analytical chemistry [48]. Each principle corresponds to an input with a score and a color range that vary from 0 (red) to 1 (dark green). The final score is obtained from the assessment of all the principles, and the result is shown in a clock-like pictogram where the score and color of the total process are in the middle. The 12 principles are represented as segments, around the middle part.

According to these, the evaluation of the proposed method obtained a score of 0.45 (Figure 5b). Although this procedure had a few drawbacks, namely, not automation and not miniaturized sample preparation, as well as the use of toxic solvents such as methanol, it could be considered as an acceptable green technique in agreement with other reported values [32].

Furthermore, the proposed extraction approach was also assessed by the AGREEprep metric, a new tool that focuses on the specific aspects related to the sample preparation technics to calculate as well as identify features that could be improved following the 10 principles of GSP [39]. The results obtained are presented in a similar way to the previously described for the AGREE metric. A colorful round pictogram with the number in the center indicates the overall sample preparation greenness performance. Around the circle, there are 10 parts, each one related to the 10 principles of GSP, with a length and color associated with the weight assigned and the performance to the respective criterion.

In this work, the AGREEprep score for the proposed method was 0.42, and although there was a lack of in situ sample preparation and automation, which were the main disadvantages of this process as in many protocols, it still represents a well-acceptable green technique.

As a summary of the three applied tools, a detailed analysis of the advantages and drawbacks of the proposed sample preparation methodology from a green perspective was achieved. As an overall result of this assessment, the PUF-based OTA removal procedure can be considered acceptable and adequate for trace analysis in herbal infusions. Additionally, this type of evaluation constitutes a novel aspect and, such as the figures of merit, would be important to be routinely considered and included in reported protocols.

## 4. Conclusions

The new updates in maximum levels of tolerance for OTA in dried herbs encourage the development of validated methods, for both surveillance and research, to quantitatively determine this mycotoxin in herbal infusion. A novel, simple, rapid, and economic extraction procedure based on the use of commercial PUF has been presented. The results obtained confirm a satisfactory recovery in herbal infusions, with suitable performance parameters such as matrix effects, linearity, sensitivity, repeatability, recovery, and accuracy. Compared to most commercial and reported SPE methods, the simple and easy preparation, the low cost, and the accessibility of PUF, added to the greenness and reliable determination of OTA, make this proposal an attractive tool for monitoring and controling OTA in infusions of wide consumption. Even though preliminary studies have confirmed the high recyclability of PUF, future works will include the evaluation of its reusability and the adaptation to automated systems in complex matrices.

## Figures and Tables

**Table 1:** Codified variables, experimental range, and levels.

Experimental Variables	Units	−1	0	1
A = sample volume	mL	3	5	7
B = sorbent mass	mg	10	30	50
C = contact time	min	10	20	30

**Table 2:** Average suppression matrix effect, recoveries, and methodological validation for commercial samples of black, green, chamomile, and mate teas.

	Black Tea	Green Tea	Chamomile Tea	Mate Tea
MEs (%, n = 3)	27	20	22	35
RE (%, n = 3)	93	87	89	78
*^d^* RSD (%)	2.8	2.7	2.2	2.5
***Figures of merit***				
*^a^* LR (ng mL^−1^)			0.5–100	
*^b^* LOD (ng mL^−1^)			0.5	
*^c^* LOQ (ng mL^−1^)			1.5	
R^2^			0.997	

*^a^* LR: linear range. *^b^* LOD: limit of detection. *^c^* LOQ: limit of quantitation. *^d^* RSD: relative standard deviation for the recovery extraction study (RE, n = 3).

## Data Availability

The data supporting this study’s findings are available from the corresponding author upon reasonable request.
